# Repeated injections of human umbilical cord blood-derived mesenchymal stem cells significantly promotes functional recovery in rabbits with spinal cord injury of two noncontinuous segments

**DOI:** 10.1186/s13287-018-0879-0

**Published:** 2018-05-11

**Authors:** Chaohua Yang, Gaoju Wang, Fenfen Ma, Baoqing Yu, Fancheng Chen, Jin Yang, Jianjun Feng, Qing Wang

**Affiliations:** 1 Department of Spine Surgery, Affiliated Hospital of Southwest Medical University, 25 Taiping Street, Jiangyang Area, Luzhou, 646000 Sichuan China; 2 Department of Pharmacy, Shanghai Pudong Hospital, Fudan University, Shanghai, 201399 China; 3 Department of Orthopaedics, Shanghai Pudong Hospital, Fudan University Pudong Medical Center, 2800 Gongwei Road, Pudong New Area, Shanghai, 201399 China

**Keywords:** Multilevel spinal injuries, Umbilical cord blood, Mesenchymal stem cells, Somatosensory evoked potentials, Balloon compression, Repeated injection

## Abstract

**Background:**

Spinal cord injuries (SCIs) are sustained by an increasing number of patients each year worldwide. The treatment of SCIs has long been a hard nut to crack for doctors around the world. Mesenchymal stem cells (MSCs) have shown benefits for the repair of SCI and recovery of function. Our present study aims to investigate the effects of intravenously infused human umbilical cord blood-derived MSCs (hUCB-MSCs) on functional recovery after subacute spinal cord compression injury of two noncontinuous segments. In addition, we compared the effects of single infusion and repeated intravenous (i.v.) injections on the recovery of spinal cord function.

**Methods:**

A total of 43 adult rabbits were randomly divided into four groups: control, single injection (SI), repeated injection at a 3-day (3RI) or repeated injection at a 7-day interval (7RI) groups. Non-immunosuppressed rabbits in the transplantation groups were infused with either a single complete dose or three divided doses of 2 × 10^6^ hUCB-MSCs (3-day or 7-day intervals) on the first day post decompression. Behavioural scores and somatosensory evoked potentials (SEPs) were used to evaluate hindlimb functional recovery. The survival and differentiation of the transplanted human cells and the activation of the host glial and inflammatory reaction in the injured spinal cord were studied by immunohistochemical staining.

**Results:**

Our results showed that hUCB-MSCs survived, proliferated, and primarily differentiated into oligodendrocytes in the injured area. Treatment with hUCB-MSCs reduced the extent of astrocytic activation, increased axonal preservation, potentially promoted axonal regeneration, decreased the number of Iba-1+ and TUNEL+ cells, increased the amplitude and decreased the onset latency of SEPs and significantly promoted functional improvement. However, these effects were more pronounced in the 3RI group compared with the SI and 7RI groups.

**Conclusions:**

Our results suggest that treatment with i.v. injected hUCB-MSCs after subacute spinal cord compression injury of two noncontinuous segments can promote functional recovery through the differentiation of hUCB-MSCs into specific cell types and the enhancement of anti-inflammatory, anti-astrogliosis, anti-apoptotic and axonal preservation effects. Furthermore, the recovery was more pronounced in the rabbits repeatedly injected with cells at 3-day intervals. The results of this study may provide a novel and useful treatment strategy for the transplantation treatment of SCI.

**Electronic supplementary material:**

The online version of this article (10.1186/s13287-018-0879-0) contains supplementary material, which is available to authorized users.

## Background

Multilevel spinal injuries are common among patients who experience traffic accidents and falls from height and require increased research efforts. Among patients with fractures/dislocations and those with spinal cord injury (SCI), 10.4% and 1.3% have been reported to have been diagnosed with multilevel injury, respectively [1], and up to 26.2% of paediatric patients with SCI to have exhibited multiple levels of damage [2]. In addition to trauma, multilevel spinal injuries can also be caused by disc herniation, ossification of the posterior longitudinal ligament, bone hyperplasia and hypertrophy of the yellow ligament. SCI results in disconnection of descending motor and ascending sensory pathways, leading to sensorimotor dysfunction and loss below the lesion. In addition, SCI induces neural cell death/apoptosis, axonal degeneration, and destruction of the microvasculature. These events trigger a subsequent cascade of pathophysiological changes (secondary events), including free radical release, astrocyte hyperplasia, excitotoxicity, haemorrhagic necrosis, mitochondrial dysfunction and inflammatory responses, resulting in delayed cellular dysfunction and death [3, 4]. The pathophysiological mechanism of multilevel SCI is more complicated than that of single segment SCI, and the consequences of multilevel SCI are often more serious.

Cell-based therapeutic approaches are an attractive and promising option for treating SCI. Grafted cells not only replace lost cells but also provide trophic support for neurons and manipulate the environment within the damaged spinal cord to facilitate axon regeneration or promote plasticity [5]. To date, various types of transplanted multipotent stem cells, including embryonic stem cells [6], neural stem cells [7] and mesenchymal stem cells (MSCs) [8, 9], have been shown to survive, differentiate and improve functional recovery after SCI. In this regard MSCs are of particular interest; cumulative evidence shows both the multipotency of MSCs [10, 11] and their ability to exert a neuroprotective effect after central nervous system (CNS) injury through the paracrine production of mitogenic, anti-apoptotic, and trophic factors [12, 13]. Among MSCs, human umbilical cord blood-derived MSCs (hUCB-MSCs) are attractive because these cells are readily available, are collected using a noninvasive method and are less immunogenic than other sources of stem cells, such as bone marrow or adipose tissue [14–16].

To date, numerous stem cell transplantation studies have demonstrated stem cell benefits, such as functional improvement, mainly in moderate acute spinal cord contusion models. However, there has been little research into engraftment efficiency in the subacute or chronic compression injury models. The microenvironment in animals with subacute or chronic compression injury is somewhat different from that in animals with acute contusions, and this difference may influence the survival and differentiation of the transplanted cells [17].

Transplantation routes are quite critical for evaluating therapeutic significance. Stem cells can be transplanted into injured CNS tissue through intralesional, intravenous (i.v.), intra-arterial or intrathecal routes [8, 18, 19]. Among these stem cell injection routes, direct injection into the injured site has been favoured due to the high engraftment percentage of transplanted cells [8]. However, intralesional transplantation (ILT) may not only further damage an already impaired spinal cord but also expose delivered cells to hostile environments [20]. Furthermore, when ILT is translated to clinical practice, stereoscopic localised puncture or major surgery is required, which could be harmful to physically debilitated patients. Therefore, more effective and minimally invasive cell delivery systems are required for transplanting cells. If we can enhance cell migration, systemic transplantation could be one of the most appropriate cell delivery routes.

Based on these considerations, we investigated the effects of i.v. injection of hUCB-MSCs on functional recovery after subacute spinal cord compression injury of two noncontinuous segments and compared single infusion and repeated i.v. injection to determine which was more beneficial for cell homing and the recovery of spinal cord function.

## Methods

### Animals

**Fig. 1:**
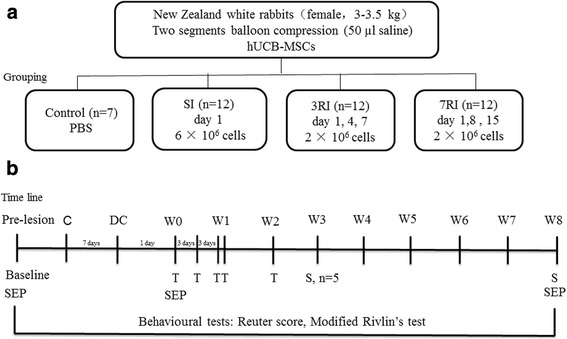
Experimental schedule. **a** The study groups. hUCB-MSCs, human umbilical cord blood-derived mesenchymal stem cells; SI, single injection; 3RI, repeated injection with 3-day intervals; 7RI, repeated injection with 7-day intervals. **b** Time line. C, compression; DC, decompression; W, weeks after the first transplantation; T, transplantation; S, sampling; SEP, somatosensory evoked potential

Adult female New Zealand white rabbits (3.0–3.5 kg, Jiagan biology, Shanghai, China) were used in this study (*n* = 43) (Fig. 1). This study was conducted in accordance with the National Institutes of Health Guide for the Care and Use of Laboratory Animals. All protocols were approved by the Institutional Animal Care and Use Committee of Fudan University Pudong Medical Centre and Affiliated Hospital of Southwest Medical University.

### Balloon compression model

Surgical techniques were performed according to a previous study from our experimental group [21]. Briefly, the animals were anaesthetised via ear vein injection of 3% sodium pentobarbital (30 mg kg^−1^). The rabbits were then placed and fixed in a prone position. The animal’s back was shaved, and a 5-cm midline incision was cut over the T4–T9 spinous processes under sterile conditions. Both the soft tissue and spinous processes of vertebrae T5–T7 were removed. Two small holes (2 mm diameter) were made in the vertebral arch of T6 and T7 using a micromotor. A groove was drilled into the midline on the dorsal surface of the T6 and T7 vertebral lamina separately to guide the insertion of the catheter and to hold it in position at the midline [22, 23]. Two 2-French Fogarty catheters (Edwards Lifesciences, CA, USA) were then inserted into the epidural space and advanced cranially or caudally for 3 cm, so that the centre of the balloon rested at approximately the T4 or T11 level of the spinal cord. Soft tissues and skin were sutured in anatomical layers, and the end of the catheter was fixed to the skin. Two balloons were inflated using 50 μl saline solution after the operation. At 7 days post compression, decompression was performed by deflating and slowly removing the catheter. Manual bladder expression was performed at least twice daily after balloon inflation until reflex bladder activity was established, and subcutaneous injections of the antibiotic enrofloxacin (10 mg/kg/day) were administered for 3 days.

### Isolation, culture, and characterisation of hUCB-MSCs

Human umbilical cord blood (UCB) was obtained from a normal, full-term pregnant woman after informed consent and ethical approval were acquired. hUCB-MSCs were isolated and expanded using a previously described protocol [24, 25]. In brief, mononuclear cells (MNCs) were separated by means of Ficoll-Hypaque (density 1.077 g/mL; GE) density gradient centrifugation at 400 g for 25 min at room temperature. MNCs were seeded at a density of 2 × 10^6^ cells/cm^2^ into 25-cm^2^ flasks containing Dulbecco’s Modified Eagle’s Medium–Low Glucose (GIBCO Invitrogen) supplemented with 10% fetal bovine serum (Gemini) and incubated at 37 °C under 5% CO_2_ in air. For the selection of MSCs, nonadherent cells were eliminated by replacing the medium 5 days after cell seeding. The medium was changed twice a week. The experiments were performed at the 3rd to 5th passage of the cultures with approximately 80–90% confluence.

To evaluate the differentiation ability of hUCB-MSCs, cells were subjected to osteogenic and adipogenic differentiation in vitro using a StemPro® differentiation kit (Gibco, Life Technologies) according to the manufacturer’s instructions. Differentiation was detected by Alizarin Red S or Oil Red O staining.

For cell-surface expression analysis, aliquots of cells at a concentration of 1 × 10^6^ cells per mL were immunolabelled at room temperature for 30 min with the following human antibodies: CD34-PE, CD73-APC, CD90-FITC, CD105-Percp-Cy5.5, and HLA-DR-PE. The corresponding controls were conjugated to fluorescein (BD Pharmingen, San Diego, CA, USA). The labelled cells were analysed by a FACSCalibur flow cytometer (Becton Dickinson) with CellQuest software.

### Transplantation of hUCB-MSCs and grouping

At 24 h post decompression, the rabbits were randomly assigned to one of the following four groups: (I) the control group that received i.v. injection of PBS (vehicle) on the transplant day (n = 7); (II) the single i.v. injection (SI) group that received 6 × 10^6^ cells at 1 day after decompression (n = 12); (III) the 3-day interval group (3RI, n = 12) that received 2 × 10^6^ cells at 1, 4 and 7 days after decompression and (IV) the 7-day interval group (7RI, n = 12) that received 2 × 10^6^ cells at 1, 8 and 15 days after decompression (Fig. 1). In the i.v. transplantation groups, hUCB-MSCs were slowly infused at a concentration of 2 × 10^6^ cells/mL through the ear vein. Immunosuppressive drugs were not administered before and after transplantation.

### Behavioural tests

Behavioural assessments were performed in a blinded fashion at baseline, on the first day after compression, before transplantation and then once weekly until euthanasia. All motor and sensory testing was conducted between 08.00 a.m. and 12.00 a.m. All animals were examined to ensure the presence of an empty bladder to avoid any influence of a full bladder on their activities.

#### Reuter score

The muscle tone, motor and sensory function of the hind legs and the reflex functions of the spinal cord were evaluated using the Reuter scoring system [26, 27] (total score of 11 points) at a predetermined time. Scores were allocated for stretch reflex as 0 (normal), 1 (slightly increased/decreased) or 2 (excited/disappeared); for pain retracting reflex as 0 (fast), 1 (slow) or 2 (disappeared); for muscular tension as 0 (normal), 1 (high/low tension) or 2 (flaccid/spastic); for back feeling as 0 (complete), 1 (partial) or 2 (disappeared) and for motor function as 0 (normal walking), 1 (able to support weight but unable to walk), 2 (spontaneous movement but unable to stand) or 3 (no spontaneous movement but responds to noxious stimulus).

#### Modified Rivlin’s test

The ability of the animals to maintain their position and the maximum angle on an inclined plane was assessed using previously described methods [28, 29]. Briefly, the animals were positioned horizontally on a custom-made oblique plate consisting of two rectangular alloy plates connected through a hinge (the test surface consisted of a rubber surface with shallow trenches), with the body axis of the rabbit perpendicular to the longitudinal axis of the oblique plate. The oblique plate was rotated around the axis starting from the horizontal position, and the maximum angle that the animals could sustain for 5 s without sliding down the plate was recorded two times for each direction and was averaged to yield a final value.

### Electrophysiological evaluations

Somatosensory evoked potentials (SEPs) were measured in seven animals to detect the functional integrity of the spinal cord. SEPs were recorded according to previously published methods used by our experimental group [21] at baseline, before transplantation (8 days post injury) and 8 weeks after the first transplantation. The animals were anaesthetised as previously described. A 3-channel Dantec-KEYPOINT electromyograph was used to generate and record SEPs. To elicit cortical SEPs, a constant current stimulator was used, with a 3-Hz square wave of 0.2 ms duration, with a pair of subdermal electrodes inserted into the median and tibial nerves of the hind limbs. The stimulation intensity was 2–3 mA, and the effectiveness of nerve stimulation was evaluated by visual inspection of the twitching of the hind limbs. Recordings from the skull electrodes were obtained at Cz–Fz. The recording electrode was located 2.5 mm posterior and 2.8 mm lateral to the bregma. Reference electrodes were placed along the midline of the forehead. A subdermal needle electrode was placed at the back of the neck as a ground. When the interference waveform was a smooth straight line, stimulation began. We averaged 600–800 SEP trials to achieve a smoother wave and to improve the signal-to-noise ratio. Sensitivity of 2 μV/div and time base of 10 ms/div was used to display the SEP responses. The SEP response was identified, and the onset latency and peak-to-peak amplitude of the response were then obtained.

### Tissue processing

The animals were deeply anaesthetised and transcardially perfused with 4% paraformaldehyde at 3 weeks (n = 5 for transplantation groups, to determine the engraftment percentage) and 8 weeks (n = 7 for each group) after the first transplantation for histological analysis. Two 1-cm sections of the spinal cord from the epicentre of the lesion at T4 and T11 were dissected and post-fixed with a 4% formaldehyde solution in phosphate buffer overnight. The samples were then dehydrated in graded ethanol solutions (75–100%) and embedded in paraffin. Sagittal sections were cut at a thickness of 4 μm and serially mounted on Superfrost Plus glass slides.

The sections were processed for immunofluorescence, followed by quantitative analysis. Tissue sections were stained with the following primary antibodies: mouse anti-human nuclear matrix antigen (hNuA, 1:100, Millipore, MAB1281) for the detection of transplanted human stem cells, rabbit anti–neuronal nuclei (NeuN, 1:100, Chemicon, ABN78) for neurons, rabbit anti-neurofilament 200 (NF200, 1:100, Sigma, N4142) for neurons and axons, rabbit anti-glial fibrillary acidic protein (GFAP, 1:1000, Millipore, AB5804) for astrocytes, rabbit anti-O1 (1:200, Chemicon, AB5991) for oligodendrocytes, rabbit anti-Ki67 (1:100, Millipore, AB9260) for proliferating cells, and mouse anti-Iba-1 (1:100, Sigma, SAB2702364) for microglia/macrophages. We used fluoro-conjugated secondary antibodies, including goat anti-rabbit/mouse Alexa Fluor 488 or 555. After nuclei were counterstained with 4,6-diamino-2-phenyl indole (DAPI), images were captured with a fluorescence microscope (Leica Instruments, Nusslosh, Germany). For TUNEL apoptotic staining, sections were rinsed, TUNEL labelled per the manufacturer’s protocol (Roche, Mannheim, Germany), and DAPI counterstained.

### Engraftment of the transplanted MSCs into the injured spinal cord

For quantitative analysis of the transplanted human stem cells in the spinal cord at 3 weeks and 8 weeks after the first transplantation, five sections spaced 120 μm apart at the epicentre of the lesion were collected from each spinal cord sample, and all hNuA and DAPI double-labelled cell profiles in each section were counted. To be counted, in addition to being labelled by both markers, each cell had to exhibit a clear cellular morphology and a discernible nucleus. Five tissue sections from the rostral or caudal spinal cord were added together to obtain a slide total. The rostral and caudal slide totals were added to obtain the total for an individual animal and the five animals constituting each group were averaged to acquire the values for each group.

### Transplanted human cell fate analysis

hUCB-MSC differentiation and proliferation were examined by double fluorescent labelling. Five animals were used for each protein marker. For quantification of each protein marker, three sections (120 μm apart) at the epicentre of the lesion were collected from the rostral and caudal spinal cord. Thus, six slices for each animal were used for fate analysis. The number of human cells and double-labelled cells (NeuN, GFAP, Olig1, Ki67) were counted using ImageJ Version 10.2 software with a cell counter plug-in. A minimum of 10 semi-random images (× 200) containing at least 10 human cells were analysed. The number of double-labelled cells is expressed as a percentage of the total number of human cells counted in each individual series. For each protein marker analysed, the values from five animals were averaged to obtain the final percentage.

### Gliosis, axonal, microglial/macrophage and apoptosis quantification

The immunofluorescence staining intensities of GFAP immunoreactivity were quantified following previously described procedures [30] (10 semi-random images (× 200) of each section at the epicentre were analysed, with a total of six slices for each animal, n = 5). The average GFAP staining intensity was obtained. Quantification of the NF-200^+^ fibres was performed in six sections per animal, which were chosen by the same approach used for the quantification of GFAP. The number of Iba-1^+^ microglia/macrophages and TUNEL^+^ cells were manually counted in 10 separate fields of view per section in the lesion centre at × 200 magnification (*n* = 5 per group).

### Statistical analysis

Statistical analyses were performed using SPSS 19.0 software (IBM, New York, NY, USA). Data are expressed as the mean ± standard deviation. The Reuter score results were analysed using the Kruskal-Wallis test, and the Rivlin’s test was analysed using two-way analysis of variance (ANOVA), followed by Bonferroni post hoc test. Data from more than two groups were analysed by one-way ANOVA, followed by Bonferroni post-hoc testing. The differences between the number of surviving cells in the rostral and caudal areas were analysed using Student’s *t* test. Differences were deemed statistically significant at *p* < 0.05.

## Results

### Functional recovery

**Fig. 2:**
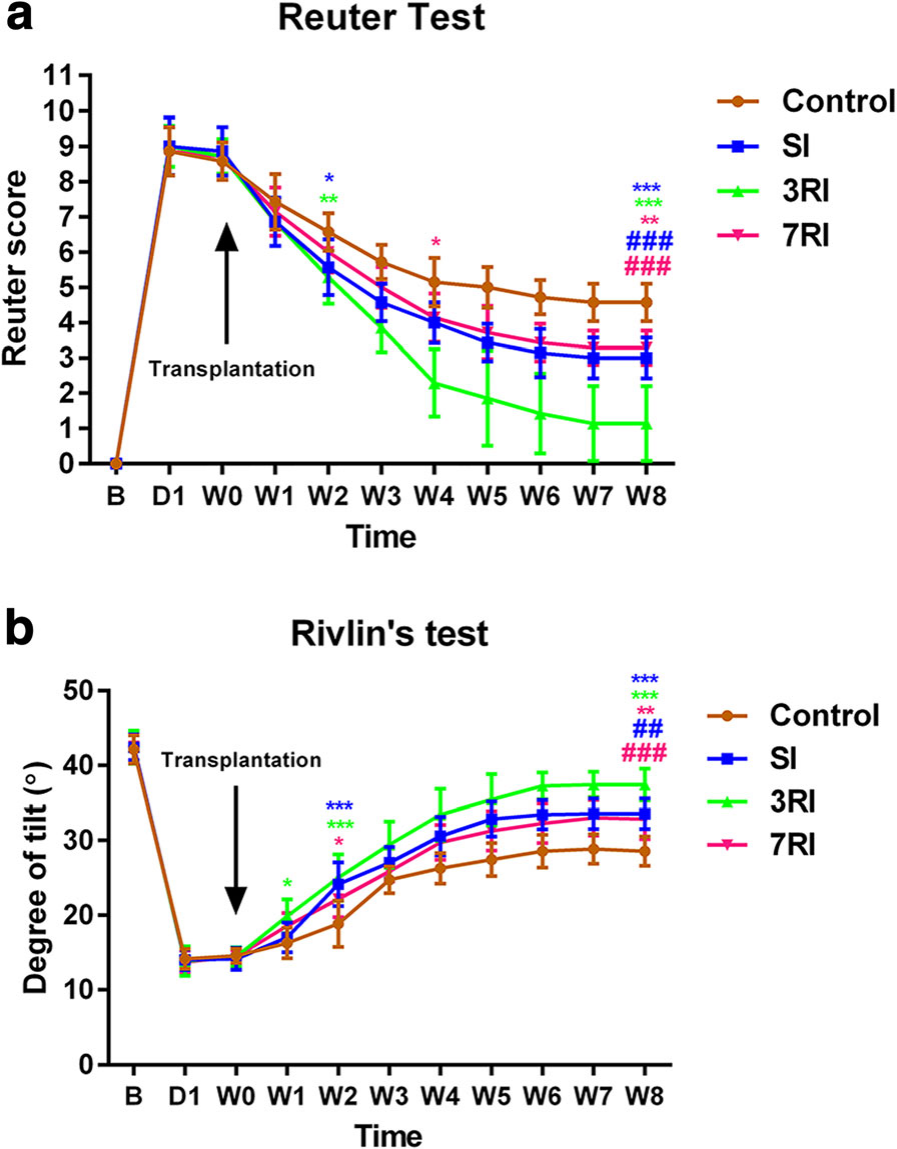
Behavioural improvement assessed by Reuter scores (**a**) and modified Rivlin’s test results (**b**) from baseline to 8 weeks after the first transplantation. *Significant differences between the transplantation and control groups (*p < 0.05, **p < 0.01 and ***p < 0.001, respectively). ^#^Significant differences for the single injection (SI) and the repeated injection at 7-day intervals (7RI) groups versus the repeated injection at 3-day intervals (3RI) group (^##^*p* < 0.01 and ^###^*p* < 0.001, respectively). **b** Baseline. D1, first day after spinal cord injury (SCI); W, weeks; W0, before transplantation

The Reuter scores and modified Rivlin’s test results of the groups obtained from baseline to 8 weeks after the first transplantation (n = 7) are shown in Fig. 2. All the injured rabbits manifested complete hind limb paraplegia at 1 day after SCI. Before transplantation (8 days post injury), rabbits with significant spontaneous recovery were excluded. There was no significant difference in the pretransplantation Reuter scores and Rivlin scores between the groups. Beginning in the 2nd week post transplantation, the Reuter scores in the SI and 3RI groups were significantly lower than those in the control group. The animals in the SI and 7RI groups had similar recovery over time. At 7 weeks after transplantation some animals in the 3RI group were able to stand and walk, and some even exhibited a normal gait. At 8 weeks post transplantation, the mean Reuter scores in the SI, 3RI, 7RI and control groups were 3.00 ± 0.58, 1.14 ± 1.07, 3.29 ± 0.49 and 4.57 ± 0.54, and the Rivlin scores were 33.57° ± 2.07°, 37.43° ± 2.15°, 32.86° ± 2.67° and 28.57° ± 1.99°, respectively. The functional recovery seen in the rabbits that underwent transplantation was significantly better than that in the control group (*p* < 0.01). The best functional recovery was observed in the 3RI group compared with the other two transplantation groups (*p* < 0.01). However, there were no differences between the SI and 7RI groups.

### Recovery of neural conduction

**Fig. 3:**
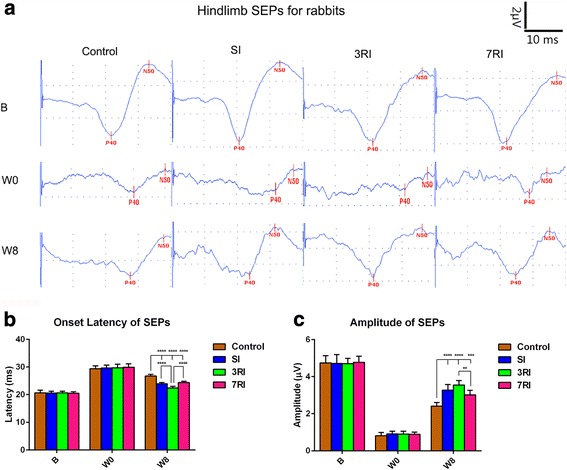
Somatosensory evoked potential (SEP) results showing functional improvements in rabbits with spinal cord injury following transplantation of human umbilical cord blood-derived mesenchymal stem cells (hUCB-MSCs) compared with rabbits subjected to PBS treatment. The best recovery of electrophysiological responses was observed in the rabbits that received repeated injections with 3-day intervals (3RI). **a** Mean SEP waveforms recorded at baseline, before the first transplantation and 8 weeks after the first transplantation from Cz–Fz during hindlimb stimulation in four groups. **b** Onset latency results at scheduled times in the four groups. **c** Peak-to-peak amplitude results at scheduled times in the four groups (**p < 0.01, ***p < 0.001 and ****p < 0.0001, respectively). SI, single injection; 7RI, repeated injections at 7-day intervals

SEPs were used to evaluate the functional integrity of ascending sensory pathways following SCI and the transplantation of hUCB-MSCs. Figure 3 indicates the changes in the SEPs of a representative animal at baseline, before the first transplantation and 8 weeks after the first transplantation. The baseline SEPs were characterised by latency after the stimulus and the peak-to-peak amplitude in all the animals. Compared with the SEPs at baseline, hindlimb SEPs had increased latency and reduced amplitude before the first transplantation. There was no significant difference between the groups in pretransplantation SEPs. At 8 weeks post transplantation, the mean onset latency was significantly shorter in the transplantation groups (SI, 23.93 ± 0.41 ms; 3RI, 22.41 ± 0.59 ms; 7RI, 24.34 ± 0.47 ms) than that in the control group (26.73 ± 0.60 ms, all *p* < 0.0001); the amplitudes of the transplantation groups (SI, 3.27 ± 0.30 μV; 3RI, 3.54 ± 0.25 μV; 7RI, 3.01 ± 0.24 μV) were significantly higher than those of the control group (2.41 ± 0.20 μV, all *p* < 0.001). The best electrophysiological recovery was observed in the 3RI group compared with the SI (latency, *p* < 0.0001, amplitude was not significantly different) and 7RI (latency, *p* < 0.0001; amplitude, *p* < 0.01) groups. However, there were no differences between the SI and 7RI groups.

### Survival and migration of transplanted hUCB-MSCs in the injured spinal cord

**Fig. 4:**
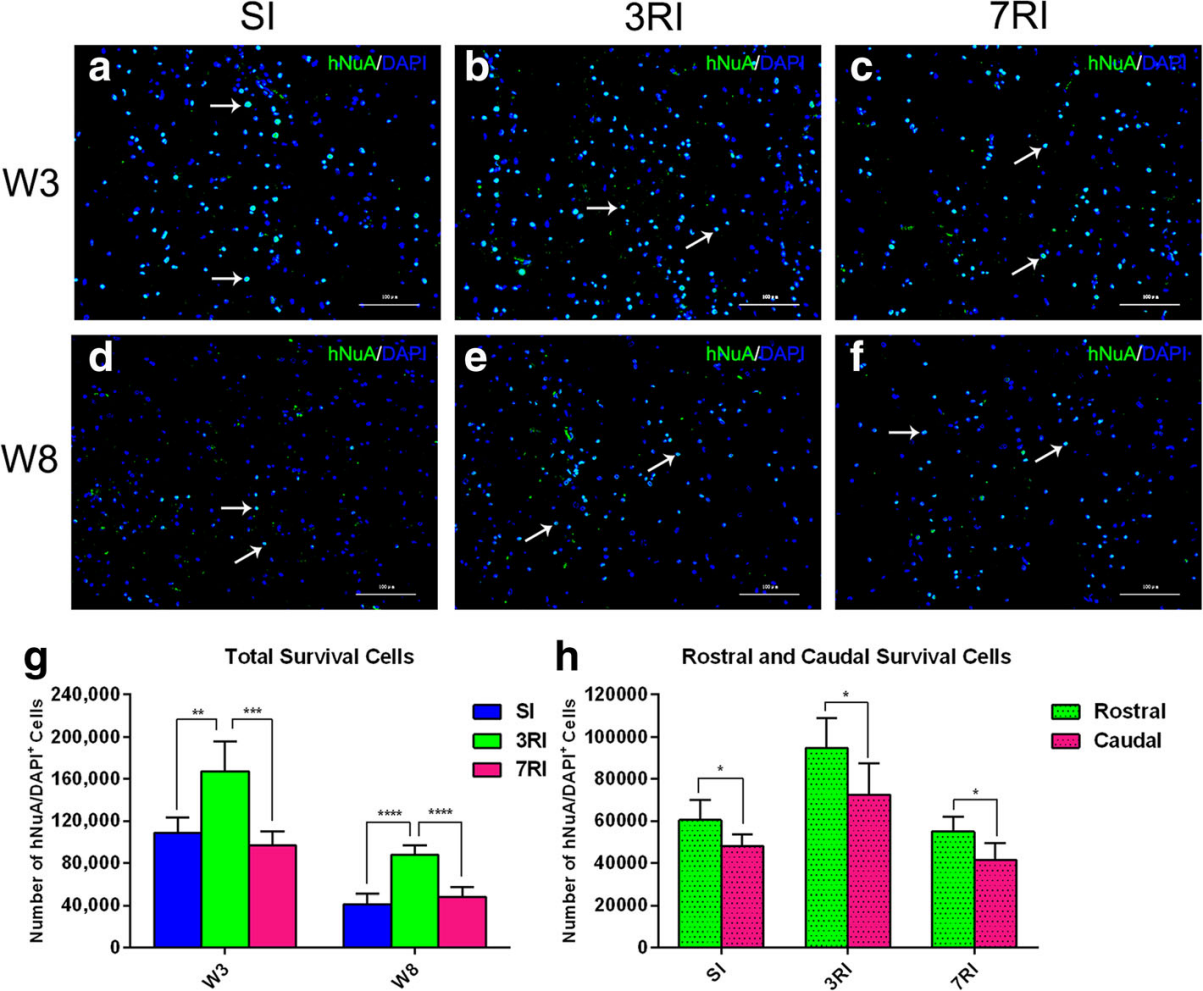
Transplanted human umbilical cord blood-derived mesenchymal stem cells (hUCB-MSCs) survive and migrate extensively in the injured spinal cord. Detection of transplanted hUCB-MSCs by specific anti-human nuclei antibody (hNuA, green) staining 3 weeks (W3) (**a**-**c**) and 8 weeks (W8) (**d**-**f**) after the first transplantation; 4,6-diamino-2-phenyl indole (DAPI) counterstaining is shown in blue. Arrows indicate a hNuA^+^/DAPI^+^ cell. **g** Quantitative analysis of the hNuA^+^/DAPI^+^ cell numbers in the single injection (SI), repeated injection at 3-day intervals (3RI) and repeated injection at 7-day intervals (7RI) groups. **h** Quantitative analysis of the hNuA^+^/DAPI^+^ cell numbers in the rostral and caudal areas of the injured spinal cord. (*p < 0.05, **p < 0.01, ***p < 0.001 and ****p < 0.0001, respectively). Scale bar = 100 μm

To investigate the survival and migration of transplanted cells (Isolation, culture, and characterization results of transplanted cells can be found in Additional file 1), we sacrificed rabbits at 3 and 8 weeks post transplantation and detected homed human cells in harvested sagittal tissue sections. Grafted human stem cells were clearly detected by immunohistochemical staining with a specific anti-hNuA antibody (Fig. 4). Homed cells were distributed throughout the grey and white matter of the injured spinal cord, and most transplanted MSCs were found around the cavitation area, with no evidence of abnormal morphology or any mass formation indicative of tumourigenesis (Fig. 4a-f). The quantitative analysis showed that the number of total surviving cells in the 3RI group (167,300 ± 28,322) was significantly higher than in the SI (108,440 ± 14,868, *p* < 0.01) and 7RI (96,680 ± 13,415, *p* < 0.001) groups at 3 weeks post transplantation. The number of total surviving cells at 8 weeks post transplantation was significantly reduced compared with the number at 3 weeks post transplantation (Fig. 4g). The number of surviving cells in the rostral area was significantly higher than that in the caudal area (*p* < 0.05) at 3 weeks post-transplantation (Fig. 4f), suggesting that the grafted cells migrate preferentially to the rostral area of the spinal cord.

### hUCB-MSCs differentiate into all three CNS cell types

**Fig. 5:**
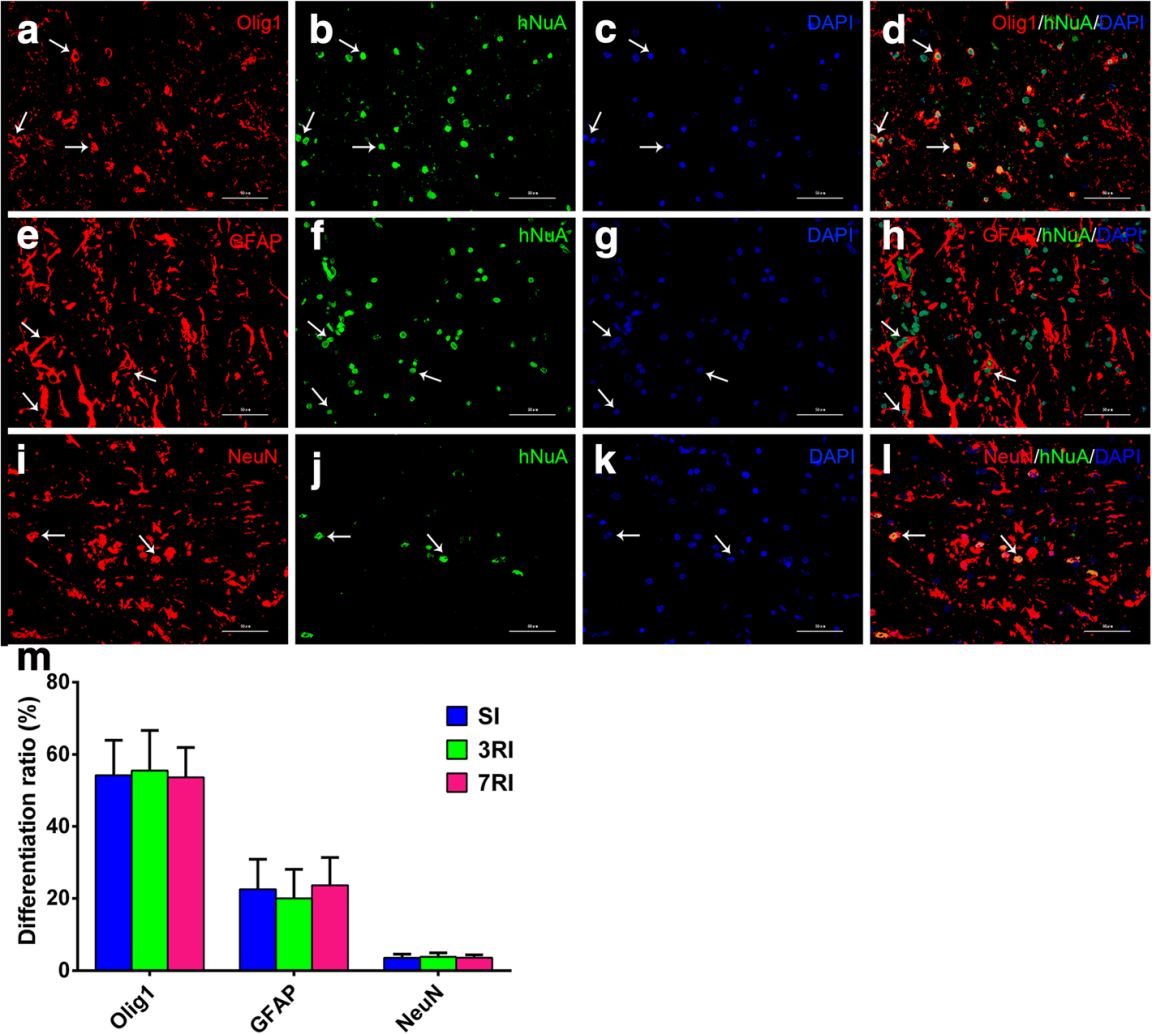
Human umbilical cord blood-derived mesenchymal stem cells (hUCB-MSCs) mostly differentiate into oligodendrocytes and astrocytes and a few neurons. **a**-**d** Oligodendrocytic differentiation of the transplanted mesenchymal stem cells (MSCs) was noted in the transplantation groups 8 weeks post-transplantation. **e**-**h** Astrocytic and **i**-**l** neuronal differentiation of the transplanted MSCs was also noted in the transplantation groups (red fluorescence for Olig1, glial fibrillary acidic protein (GFAP), and NeuN antibody, green fluorescence for hNuA antibody, blue for 4,6-diamino-2-phenyl indole (DAPI)). Arrows indicate a double-labelled cell. **m** Quantitative analysis of the Olig1^+^/hNuA^+^, GFAP^+^/hNuA^+^, and NeuN^+^/hNuA^+^ cell numbers in the single injection (SI), repeated injections at 3-day intervals (3RI) and repeated injections at 7-day intervals (7RI) groups. The results show that there was no significant difference in the differentiation ratio of human stem cells among the transplantation groups 8 weeks post transplantation. Scale bar = 50 μm

Histological examination revealed that hUCB-MSCs survived for 8 weeks after transplantation. To investigate whether transplanted cells promote regeneration via replacement mechanisms, we assessed the fate and differentiation of hUCB-MSCs by double immunofluorescence in spinal cords that were sectioned sagittally from a subset of hUCB-MSC-transplanted animals (Fig. 5). The quantification results revealed that 54.5 ± 9.1% of human cells exhibited differentiation along the Olig1-positive oligodendrocyte lineage, which was the predominant fate of transplanted hUCB-MSCs. GFAP-positive astrocytes accounted for 22.1 ± 7.6% of differentiated human cells. However, only a few hNuA immunopositive cells differentiated into NeuN-positive neurons, which accounted for 3.7 ± 0.9%. There was no significant difference in the differentiation ratio of human stem cells among the transplantation groups at 8 weeks posttransplantation (Fig. 5m).

**Fig. 6:**
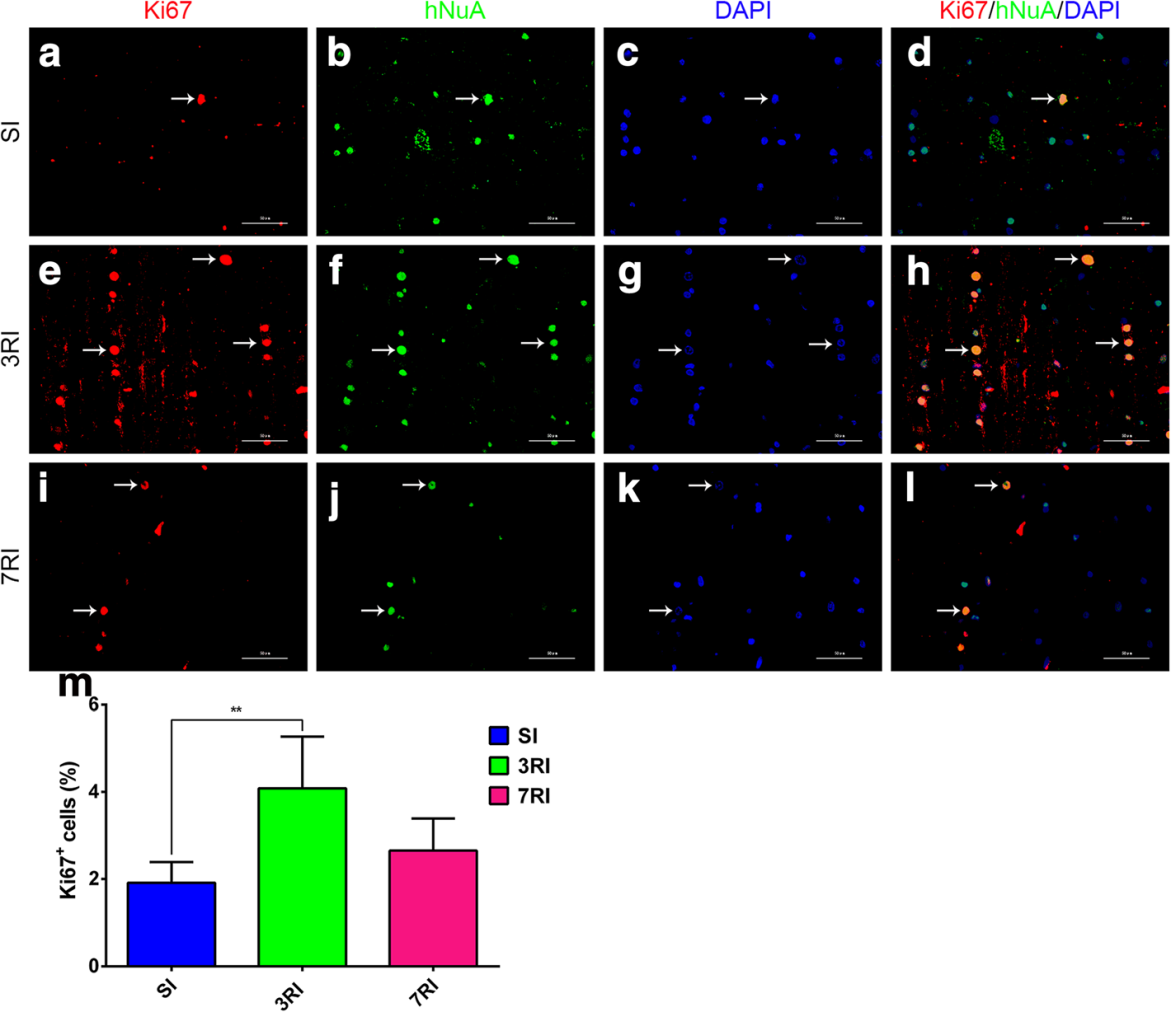
Human umbilical cord blood-derived mesenchymal stem cells (hUCB-MSCs) express Ki67 8 weeks following transplantation. Human nuclei antibody-positive cells (hNuA^+^), which are shown in green (**b**, **f**, **j**), were rarely associated with the cell cycle marker Ki67, which is shown in red (**a**, **e**, **i**); 4,6-diamino-2-phenyl indole (DAPI) counterstaining is shown in blue (**c**, **g**, **k**) in the single injection (SI) (**a**-**d**), repeated injections at 3-day intervals (3RI) (**e**-**h**) and repeated injections at 7-day intervals (7RI) (**i**-**l**) groups. Arrows indicate double-labelled cells. **m** Quantitative analysis of the Ki67^+^/hNuA^+^ cell numbers in the SI, 3RI and 7RI groups. The results show that the percentage of Ki67 in the 3RI group was greater than that in the SI group (**p < 0.01). Scale bar = 50 μm

Furthermore, we investigated whether human stem cells exhibited active cell division/proliferation by double labelling for hNuA and Ki67 (Fig. 6). Most grafted cells did not express Ki67 (1.9 ± 0.4%, 4.1 ± 1.2% and 2.7 ± 0.7% in the SI, 3RI and 7RI groups, respectively, n = 5 for each group), suggesting limited proliferation of hUCB-MSCs at 8 weeks post transplantation. There was a significant difference in the percentage of Ki67^+^ cells between the SI group and the 3RI group (*p* < 0.01) (Fig. 6m). Ki67-positive cells were randomly dispersed across the injured area without evidence of clustering at specific sites.

### hUCB-MSCs attenuated gliotic scarring and promoted axonal preservation

**Fig. 7:**
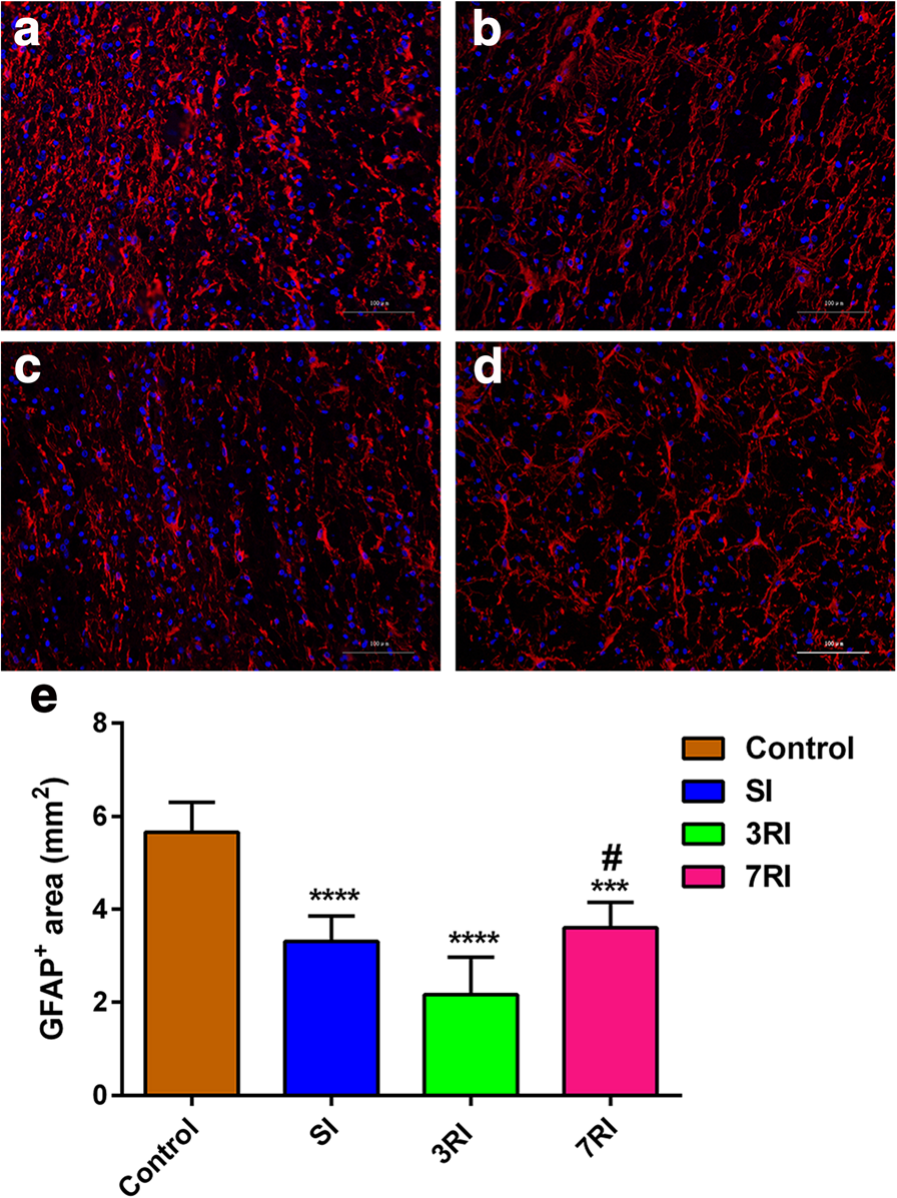
Immunofluorescence analysis of glial scarring. Representative images of glial fibrillary acidic protein (GFAP) immunostained longitudinal sections from animals with PBS control (**a**), single injection (SI) (**b**), repeated injections at 3-day intervals (3RI) (**c**) and repeated injections at 7-day intervals (7RI) (**d**) treatment that were sacrificed 8 weeks after transplantation. **e** Quantification of GFAP intensities (***p < 0.001, ****p < 0.0001 versus the control group; ^#^*p* < 0.05 versus the 7RI group). Scale bar = 100 μm

Gliosis was quantified by measuring the density of GFAP immunoreactivity. In the spinal cord tissue sections of the control group, dense GFAP immunoreactivity was detected surrounding the lesion centre at 8 weeks post transplantation, and these astrocytes were packed tightly together, forming a scar barrier. In contrast, astrocytic fronts were less prominent, and GFAP immunoreactivity in the adjacent regions was much weaker in the 3RI group than in the control and 7RI groups. Quantification showed that GFAP staining intensities were significantly reduced by treatment with hUCB-MSCs (all *p* < 0.001) and that the extent of the decrease caused by treatment measures with 3RI was significantly greater than that caused by 7RI (*p* < 0.05) (Fig. 7).

**Fig. 8:**
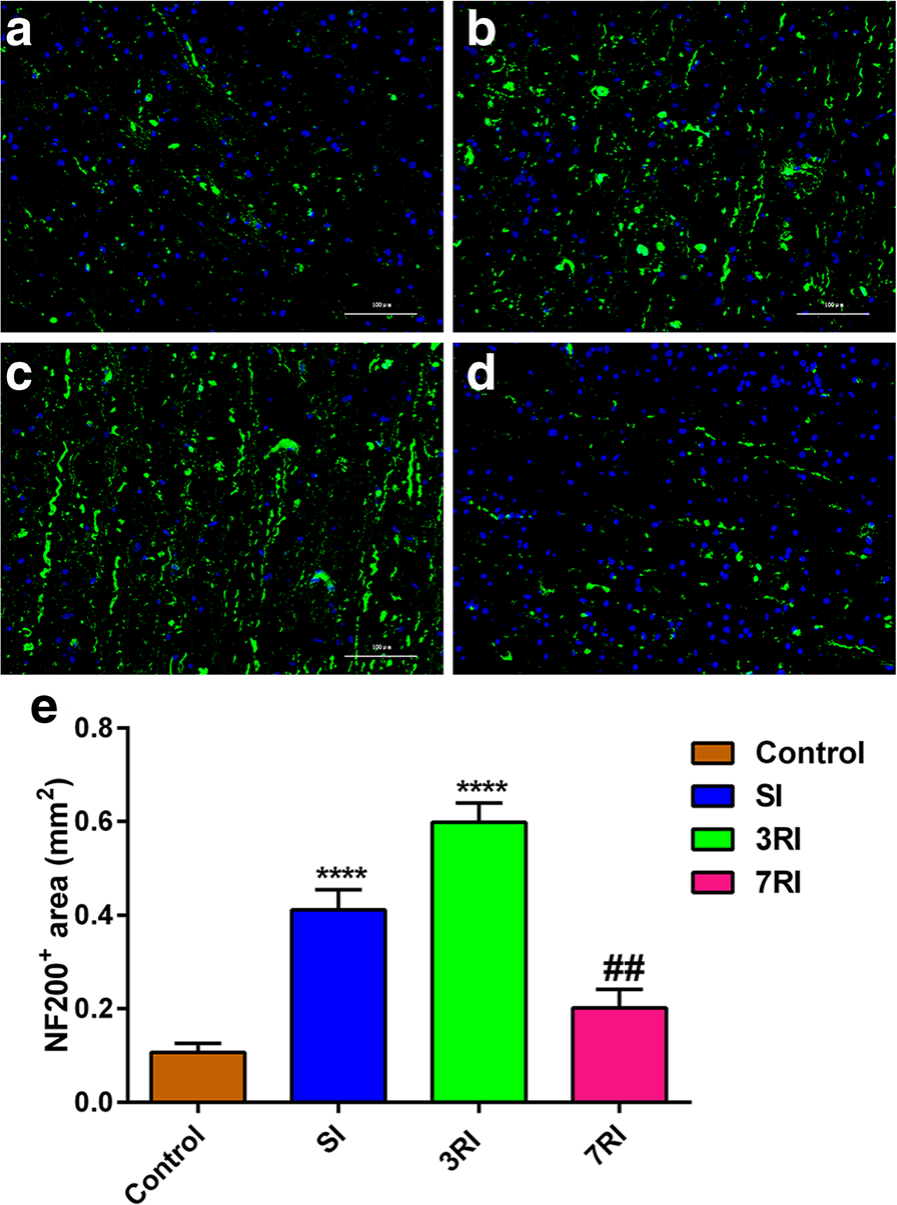
Axonal staining in the injured spinal cord 8 weeks post transplantation. Immunoreactive staining in the control group (**a**), single injhection (SI) group (**b**), repeated injections at 3-day intervals **(**3RI) group (**c**) and repeated injections at 7-day intervals **(**7RI) group (**d**) and quantification (**e**) of NF-200^+^ fibres in the epicentre of the lesion (****p < 0.0001 versus the other three groups, ^##^*p* < 0.01 versus the control group). Scale bar = 100 μm

To examine whether treatment with hUCB-MSCs affected the preservation of neurofilaments, we performed immunofluorescence analysis with an anti-neurofilament H (NF-200) mAb at 8 weeks post transplantation (Fig. 8). Compared with the control group, the stem cell transplantation group exhibited greater preservation of NF-200^+^ axons in the lesion area (*p* < 0.01). The maximum preservation of NF-200^+^ axons was observed in the 3RI group (all *p* < 0.0001) (Fig. 8e).

### hUCB-MSCs inhibited microglial/macrophage infiltration and apoptosis

**Fig. 9:**
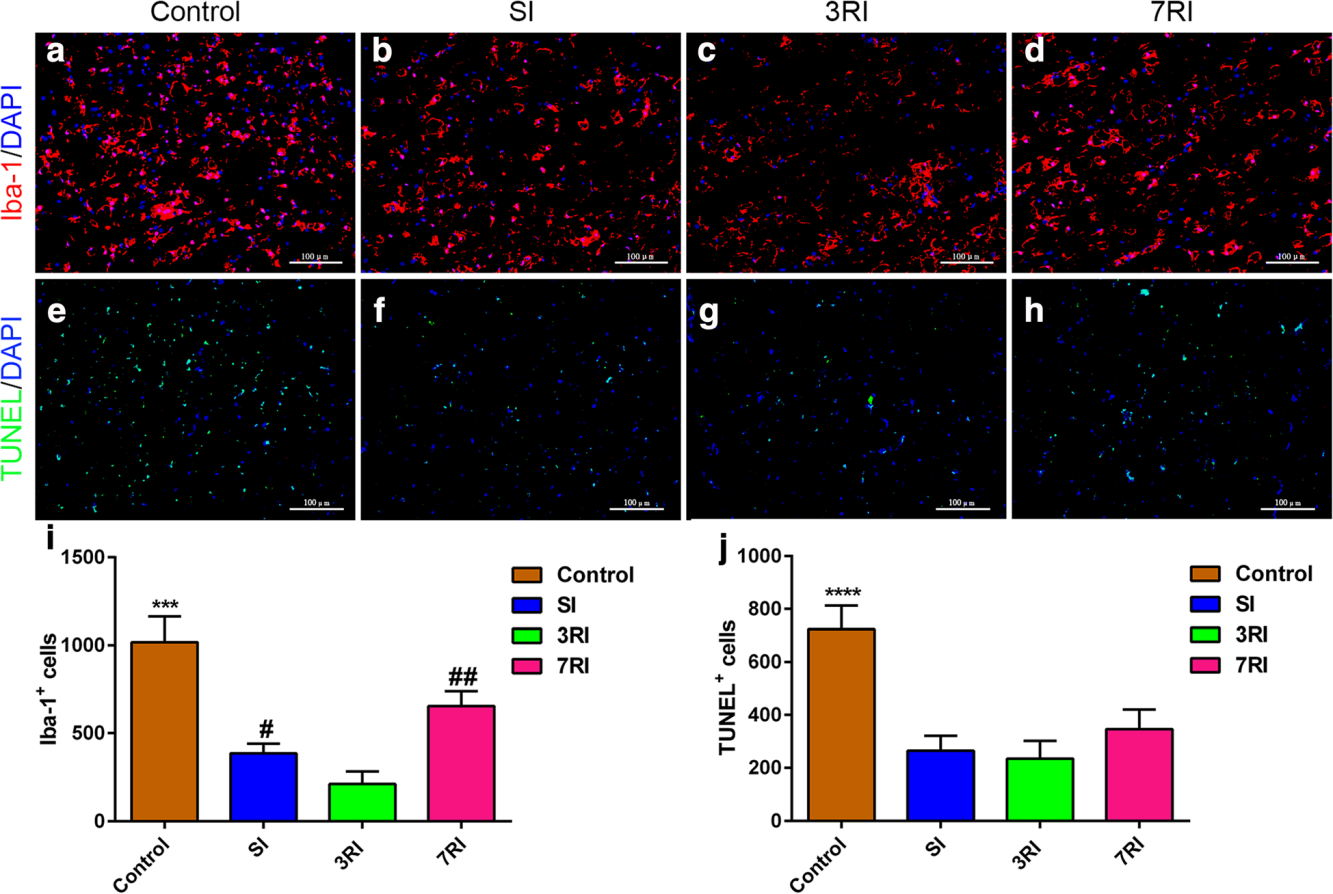
Immunofluorescence analysis of inflammation and apoptosis. Representative images of Iba-1 (**a**-**d**) and TUNEL (**e**-**h**) immunostained longitudinal sections from the control, single injhection (SI), repeated injections at 3-day intervals **(**3RI) and repeated injections at 7-day intervals **(**7RI) groups. Quantitative analysis of the Iba-1^+^/4,6-diamino-2-phenyl indole (DAPI)^+^ (**i**) and TUNEL^+^/DAPI^+^ (**j**) cell numbers in the epicentre of the lesion (***p < 0.001, ****p < 0.0001 versus the other three groups; ^#^*p* < 0.05, ^##^*p* < 0.01 versus the 3RI group). The results show that human umbilical cord blood-derived mesenchymal stem cells (hUCB-MSC) transplantation inhibited microglial/macrophage infiltration and apoptosis. Scale bar = 100 μm

Histological quantification of inflammatory infiltration and apoptotic cells was performed at 8 weeks post-transplantation. In this study, Iba-1 was used as a marker for the activation of resident microglia and extravasated macrophages (Fig. 9a-d), while TUNEL staining was used to confirm the apoptotic cells (Fig. 9e-h). The results showed that Iba-1^+^ and TUNEL^+^ cells were scattered throughout the SCI area. The average numbers of Iba-1^+^ cells in the SI, 3RI, 7RI, and control groups were 385 ± 55, 210 ± 71, 654 ± 84, and 1018 ± 145, respectively (n = 5). The number of Iba-1^+^ cells in the rabbits that underwent stem cell transplantation was significantly lower than that in the control group (*p* < 0.001). Additionally, the minimum number of Iba-1^+^ cells was observed in the 3RI group compared with the other three transplantation groups (all *p* < 0.05) (Fig. 9i).

An analysis of TUNEL^+^ apoptotic cell numbers showed that the hUCB-MSC transplantation groups had lower counts than the control group (all *p* < 0.0001) (Fig. 9j). However, there were no differences in TUNEL^+^ cell numbers among the three transplantation groups (control, 723 ± 89; SI, 265 ± 57; 3RI, 234 ± 68; 7RI, 346 ± 74). These results support the idea that hUCB-MSC transplantation inhibited microglial/macrophage infiltration and apoptosis in rabbits subjected to spinal cord compression injury of two noncontinuous segments.

## Discussion

To our knowledge, the current study is the first to specifically examine the effects of systemic injection of hUCB-MSCs in non-immunosuppressed rabbits with subacute spinal cord compression injury of two noncontinuous segments. As with a single segmental SCI, hUCB-MSCs also promoted functional locomotor recovery after transplantation into a model of subacute SCI of two segments, according to the Reuter scores and modified Rivlin’s behavioural test results. Furthermore, functional recovery in the 3RI group was significantly better than that in the SI and 7RI groups. The observed functional improvement correlates well with reduced gliotic scarring or increased axonal preservation and inhibited microglial/macrophage infiltration and apoptosis. To achieve these beneficial effects, hUCB-MSCs successfully homed, engrafted, proliferated and primarily differentiated into oligodendrocytes at the lesion site.

### hUCB-MSCs promote functional recovery

hUCB-MSCs are considered a promising therapy for the treatment of SCI [31]. hUCB-MSCs grafted into single segmental SCI models result in variable degrees of functional improvement in different established animal models. Park reported that direct injection of 3 × 10^5^ hUCB-MSCs into a contusion SCI rat model significantly promoted functional recovery 2 weeks after grafting, and it continued for up to 6 weeks [32]. Another study by Cui demonstrated that the transplantation of 5 × 10^4^ hUCB-MSCs after contusive SCI significantly promoted functional recovery 2–4 weeks after the graft [33]. In the present study, we examined the treatment effects of i.v. injection of hUCB-MSCs in rabbits after subacute spinal cord compression injury of two noncontinuous segment. The results showed that significant functional recovery began 2 weeks after the graft and continued for up to 8 weeks in the hUCB-MSC-transplanted animals compared with the control group using Reuter scores and modified Rivlin’s test (Fig. 2). Surprisingly, significant Rivlin’s score improvements occurred in the 3RI group as early as 1 week after the graft (Fig. 2b), which might have been caused by reduced inflammation, modification of the microenvironment or prevention of apoptosis [34–37]. Additionally, some animals in the 3RI group were able to stand and walk 8 weeks after the graft, and some even had nearly normal gait; the Reuter scores and Rivlin’s scores in these rabbits were improved up to 0 and 40°, respectively. In agreement with the present findings, a previous study by Lee and Cui reported near normal locomotor function recovery after grafting [31, 33]. These results suggest that hUCB-MSCs are beneficial in different SCI models, including single segment contusion or two segment compression injury models.

Discussion of the additional advantages of repeated i.v. administration of stem cells is controversial in the literature. Song et al. [38] reported that repeated i.v. administrations of human neural stem cells (hNSCs) at 7-day intervals after stroke offer no advantages over single administration. Another study, which investigated human bone marrow-derived mesenchymal stromal cell therapy in a rat model of cavernous nerve injury, demonstrated that repeated treatments at 14-day intervals did not provide any benefit for the recovery of erectile function and histomorphometric changes [39]. However, many other investigators reported that repeated administration of MSCs was more beneficial for liver failure (7-day intervals) [40], contusive SCI (3-day intervals) [41] and could improve radiation-induced proctitis in pigs by reducing inflammation and fibrosis (7-day intervals) [42]. Additionally, Jang reported that hUCB-MSCs significantly prevented graft-versus-host disease (GVHD) only when repeatedly injected at 3-day intervals [43]. Based on this information, we hypothesised that the interval time might be the key factor affecting the outcome of transplantation. Therefore, in this study, hUCB-MSC transplantation was performed once or repeated at 3-day and 7-day intervals. Our results indicated that functional recovery in the 3RI group was significantly better than that in the SI and 7RI groups. However, there were no differences between the SI and 7RI groups, which is consistent with the previous findings [38, 39]. Our results encourage and support the notion that the interval time might be the key factor affecting the outcome of transplantation [44].

### hUCB-MSCs promote sensory pathway recovery

SEPs, which are commonly used for intra-operative monitoring of the spinal cord [45, 46], have been introduced for the diagnosis, prognosis and quantification of the physiological integrity of the sensory pathways [47, 48], which extend from the periphery to the somatosensory cortex. A substantial number of studies have confirmed that SEPs may serve as a biomarker of neurological status [49, 50] and indicators of ultrastructural damage [51], with high sensitivity and specificity for the detection of electrical conductivity of the dorsal ascending pathways [52].

In the present study, the SEP latency of all groups initially increased before transplantation (8 days post injury). This increase reflects the decrease in conductivity that results after injury due to damage to both the axons and their surrounding insulating myelin. Following hUCB-MSC transplantation, the latency of the hUCB-MSC treatment groups was significantly shorter than that of the control group by week 8 (Fig. 3). Surprisingly, the latency of some animals in the 3RI group decreased to normal baseline values by week 8 (Fig. 3a). Therefore, the presence of transplanted hUCB-MSCs may facilitate the remyelination of intact axons and restore their conductivity. This change could occur by increased conductivity via remyelination of spared axons that would then result in decreased SEP latency.

Consistent with the changes in SEP latency, the amplitudes of the hUCB-MSC transplantation groups were affected by the applied treatment. Our results showed that SEP amplitudes in the transplantation groups were significantly higher than those in the control group 8 weeks after transplantation (Fig. 3). Therefore, the presence of transplanted hUCB-MSCs may increase the sparing of intact axons. This would be suggested by the increased absolute number of intact, simultaneously excitatory axons and could result in increased SEP amplitudes [53]. Although SEP latency recovered to normal baseline values in the 3RI group, SEP amplitudes in these animals remained below baseline values. This finding is consistent with the persistence of the injury-induced cavity, which remains in treated animals and prevents any severed axons from re-growing across the lesion [53].

Additionally, the SEP results indicated that repeated transplantation at 3-day intervals was more conducive to the recovery of spinal sensory pathways than a single transplantation and transplantation at 7-day intervals (Fig. 3).

### hUCB-MSCs successfully home, engraft, proliferate and differentiate

According to previously published papers, the i.v. transplantation route has a lower rate of engraftment in CNS injury models than intralesional and lumbar puncture routes or the intracisternal transplantation route [10, 18, 19, 54], which may be related to the first-pass effect of i.v. transplantation, i.e., filtering by the spleen, lungs and liver [55]. However, compared with other transplantation routes, i.v. grafts with an enhanced cell homing effort could be an effective transplantation route due to the minimally invasive and easy operation.

Here, we attempted to promote stem cell homing by repeat transplantation and adjustment of the time interval of the grafts. As expected, our results showed that hUCB-MSCs successfully migrated to the lesion site. Notably, the engrafted cells avoided the lesion and occupied the spared tissue around the lesion; the engraftment of the cells in the 3RI group (167,300 ± 28,322) was significantly greater than that in the SI (108,440 ± 14,868) and 7RI (96,680 ± 13,415) groups at 3 weeks post graft (Fig. 4g). This difference might be related to the timing of xenografting of stem cells in the peripheral circulation and stem cell migration factors. In an Alzheimer’s disease-like murine model, Ehrhart reported that no hUCB-MSCs were recovered in the peripheral circulation, even at 24 h post-transplantation [56]. This result indicated that stem cells in peripheral blood are removed very quickly, which suggests that a large number of stem cells from a single transplant might be filtered by the spleen, lungs and liver before homing to the lesion site. Additionally, previous studies have shown that some chemoattractants, such as tumour necrosis factor-alpha (TNF-α), hepatocyte growth factor (HGF), stromal-derived growth factor-1 (SDF1) (secreted from tissues around injury and inflammation sites) and brain-derived neurotrophic factor (BDNF), can efficiently stimulate the migration of implanted MSCs [57–59]. Furthermore, implanted MSCs also release HGF, SDF1 and BDNF [59, 60]. Thus, repeated transplantation at 3-day intervals might have produced a cascade of amplification reactions to recruit a large number of MSCs. However, possibly due to the short half-life of chemoattractants, the engraftment of cells in the repeated transplantation in the 7-day-interval group was lower than that in the 3-day-interval group.

Several studies on SCI models have reported that most of the administered cells disappear from the lesion site within 1 month [61, 62]. Quertainmont et al. found no intravenously grafted MSCs in lesion sections examined as early as 7 days post surgery, although neurological improvements were seen after 1 month [61]. Therefore, we killed five rabbits from the transplantation groups at 3 weeks after onset of the grafts to detect intralesional cells. We killed the remaining rabbits 5 weeks later to detect neurological recovery. The presence of hUCB-MSCs was confirmed in spinal cord sections obtained at both time points (3 and 8 weeks after grafts) in this study. Although the cell numbers were dramatically reduced 8 weeks after the grafts, a large number of transplanted cells were still detected in the transplantation groups. Different studies have reported rapid loss of donor cells within 1–6 weeks following SCI [9, 18, 63], while others have reported prolonged survival of transplanted donor cells, ranging from 2 weeks to 4 months [35]. Although this difference is puzzling, there are some differences in the injury models used in each of these studies and in the cell types used. Surprisingly, in this study, we did not use immunodeficient animals or anti-rejection drugs (cyclosporine A), but we still detected a large number of the xenografted hUCB-MSCs at the lesion site in the rabbits 8 weeks after grafting. This result indicates that immunosuppression does not improve donor cell survival [9]; additionally, it also reflects the strong immune regulation [64] and low immunogenicity [14] of hUCB-MSCs.

Interestingly, in agreement with previous findings [65], more cells were found rostrally than caudally (Fig. 4h), which may be due to the fact that the rostral cord still received connections from the brain, possibly contributing to a more trophic environment for transplanted cells. Further studies to elucidate the molecular mechanisms that result in more cells homing to rostral spinal cord are necessary and ongoing in our laboratory. These investigations will be helpful to further understand the factors that impact stem cell homing.

Regarding the fate of the transplanted cells, differentiation into glial and neuronal lineages was noted to a broader extent in our study. As shown by our double immunofluorescence analysis, we found that the majority of hUCB-MSCs differentiated along the oligodendrocyte lineage expressing the immature Olig1 marker (54.5 ± 9.1%), while a small number of cells differentiated into GFAP-positive astrocytes (22.1 ± 7.6%), and only approximately 3.7 ± 0.9% of transplanted cells differentiated into NeuN-positive neurons. Although MSC differentiation along glial or neuronal pathways has been reported by some groups [10, 11, 66], many others have been unable to detect any neural or glial lineage differentiation [18, 30, 67]. The authors suggest that functional recovery induced by transplanted MSCs might occur through modification of the CNS lesion milieu. In our results, we identified some glial and neuronal differentiation. However, oligodendrocyte phenotype expression was predominant. This result somewhat conflicts with other reports [10, 68, 69]. Although this difference is puzzling, there are some differences in the injury models used in each of these studies, the timing of cell transplantation and during differentiation, the cell types used and manipulation of cells. Salazar reported that the cell types (neutrophils, macrophages/microglia and T cells) that are present in the spinal cord microenvironment at the time of cell transplantation and during differentiation may influence the fate of transplanted cells [65]. Furthermore, oligodendrocyte differentiation may facilitate the remyelination of intact axons and restore their conductivity, resulting in shortened SEP latency. Moreover, these findings suggest that more than one cell type (for example: neurons and oligodendrocyte) may be important for facilitating sensory and motor function recovery after SCI.

Finally, a small percentage of cells expressed Ki67 (Fig. 6m) at the terminal time point, indicating that some limited continuing proliferation occurs at 8 weeks post graft. Notably, the percentage of Ki67-positive cells in the 3RI group was higher than that in the SI group. Further studies to elucidate the potential molecular mechanisms that result in more cell proliferation in the 3RI group are necessary and ongoing in our laboratory. However, no evidence of excessive proliferation, clusters of proliferating cells or tumour formation was observed in any transplanted animal.

### hUCB-MSCs attenuated gliotic scarring and promoted axonal preservation

Glial scars are thought to be a major impediment for axon regeneration following SCI. Hypertrophic astrocytes pack together tightly to form a scar barrier and express inhibitory molecules, such as chondroitin sulfate proteoglycans (CSPGs) [30]. Hence, in the present study, we investigated the glial scar area to determine whether hUCB-MSCs reduced the area of the scar. Alternatively, hUCB-MSCs that differentiated into astrocytes could have exacerbated the host glial scar. The quantification of these parameters revealed that compared with the control group, hUCB-MSC transplantation significantly reduced gliosis around the injury site 8 weeks post transplantation (Fig. 7). Consistent with our results, different studies have reported that treatment with MSCs can attenuate gliotic scar formation following SCI [30, 34, 70], while others have reported that hNSCs did not affect the glial scar area [65]. This difference might be due to the cell type used and the time point (acute or chronic stage) of cell transplantation following SCI. A previous study reported that ex vivo delivery of HGF (secreted by MSCs, as previously discussed) markedly diminished transforming growth factor-β (TGF-β, a key signal in glial scarring [71]) levels and attenuated astrocytic scar formation in an in vivo SCI model [72]. Veeravalli also suggested that hUCB-MSC treatment after SCI could upregulate matrix metalloproteinase-2 (MMP-2) and reduce the formation of the glial scar, thereby creating an environment suitable for endogenous repair mechanisms [34]. Therefore, we speculated that stem cell transplantation might diminish glial scar formation by the aforementioned mechanism, but it could not attenuate the glial scar that had already formed. Our results support the idea that hUCB-MSC transplantation after SCI mediates functional recovery not only by cellular integration with the host but also by microenvironment modification of the host.

Furthermore, as expected, the hUCB-MSC transplantation groups had more NF-200^+^ axons in the lesion area than the control group (Fig. 8). The underlying mechanisms of transplantation of cells may include secretion of neurotrophic factors and anti-inflammatory factors that reduce axonal injury, resulting in more surviving axons, which may also create an environment, as we previously discussed, suitable for axonal regeneration. Moreover, the reduced fibrotic scarring and preserved axons were more obvious in the 3RI group compared with the SI and 7RI groups. Perhaps hUCB-MSC transplantation with 3-day intervals results in more surviving cells that integrate with host tissue (Fig. 4), thus secreting more HGF and MMP-2.

### hUCB-MSCs inhibited microglial/macrophage infiltration and apoptosis

The inflammatory process caused by injury to the CNS, including the infiltration/activation of microglia and macrophages in the injured spinal cord and the production of proinflammatory cytokines and related immune effector molecules that can induce both necrotic and programmed cell death, results in deleterious neurological deficits [73]. There is substantial evidence suggesting that MSCs could reduce the inflammatory process in the injured CNS. Liao and Zhang reported that MSCs can modulate the activity of microglia, inducing these cells to release growth factors and/or anti-inflammatory cytokines [74, 75]. Urdzíková suggested that grafted MSCs can modify the inflammatory environment by shifting macrophage phenotypes from proinflammatory M1 to anti-inflammatory M2 and by reducing the levels of TNF-α and other inflammatory cytokines [70]. In accordance with these results, our study showed that vein-grafted hUCB-MSCs significantly inhibited the infiltration of Iba-1^+^ microglia/macrophages and reduced the number of TUNEL^+^ apoptotic cells. These results were associated with enhanced stem cell survival in the injured spinal cord. Grafted hUCB-MSCs were clearly detected by immunofluorescence staining using a specific anti-hNuA antibody. Our results, along with evidence that transplanting these cells leads to an improvement in locomotor and sensory function, suggested that a reduction in inflammatory activity after hUCB-MSC transplantation provides a favourable microenvironment for cell survival and tissue repair, which can promote functional recovery following SCI. Moreover, our study showed that anti-inflammatory and apoptosis inhibitory effects were more obvious in the 3RI group compared with the SI group. It appears that repeated applications at 3-day intervals may prolong the beneficial effects induced by MSC administration [70], including the reduction of the inflammatory reaction and apoptosis.

## Conclusion

As with single segmental SCI, transplanting hUCB-MSCs into subacute SCI of two segments also promotes functional outcomes as measured by behavioural tests, electrophysiological tests and immunostaining. We found that hUCB-MSCs intravenously transplanted into non-immunosuppressed SCI rabbits survived, proliferated and differentiated primarily into oligodendrocytes. Furthermore, hUCB-MSCs improved functional recovery via reduction of the inflammatory reaction and apoptosis, promotion of axonal preservation and modulation of glial scar formation. In addition, the engraftment of hUCB-MSCs was greater in rabbits subjected to repeated transplantation at 3-day intervals than that in rabbits subjected to single or repeated transplantation at 7-day intervals, which resulted in a greater therapeutic effect following transplantation at 3-day intervals. Furthermore, the beneficial effects induced by MSC application may be prolonged by repeated applications of the cells at optimal time intervals. Based on the remarkable functional improvement without adverse effects [76–78] observed in SCI and the minimally invasive and easy operation, repeated i.v. transplantation at 3-day intervals appears to be a novel and useful treatment strategy for clinical application to CNS injuries.

## Additional file

Additional file 1: Supplementary material for this article about isolation, culture, and characterization results of hUCB-MSCs can be found at Stem Cell Research & Therapy online. (DOCX 1028 kb)

## Abbreviations

3RI: Repeated injection with 3-day intervals
7RI: Repeated injection with 7-day intervals
CNS: Central nervous system
DAPI: 4,6-Diamino-2-phenyl indole
GFAP: Glial fibrillary acidic protein
hUCB-MSCs: Human umbilical cord blood-derived mesenchymal stem cells
ILT: Intralesional transplantation
i.v.: Intravenous
MNC: Mononuclear cell
MSC: Mesenchymal stem cell
PBS: Phosphate-buffered saline
SCI: Spinal cord injury
SEP: Somatosensory evoked potential
SI: Single injection
