# Supplementary material for: Repeated injections of human umbilical cord blood-derived mesenchymal stem cells significantly promotes functional recovery in rabbits with spinal cord injury of two noncontinuous segments
Source: Stem Cell Res Ther. 2018 May 11;9:136. doi: 10.1186/s13287-018-0879-0 (PMC5948759; doi:10.1186/s13287-018-0879-0)
Supplement: Supplementary file 1 — Supplementary material for this article about isolation, culture, and characterization results of hUCB-MSCs can be found at Stem Cell Research & Therapy online. (DOCX 1028 kb) [file 13287_2018_879_MOESM1_ESM.docx]

**Isolation, culture, and characterization of hUCB-MSCs**

The MNCs that were isolated from hUCB consisted of the two following types of cells: a small number of spindle-like cells and a large number of osteoclast-like cells. Osteoclast-like cells were large, round or oval-shaped and possessed multiple nuclei. The majority of the spindle-like cells were MSCs, which were successfully isolated from 20 of the 36 samples of hUCB; however, only nine samples were amplified and cultured in vitro.

In the early stages of culture, hUCB-MSCs were round. After 10 days in culture, adherent cells exhibited bipolar fibroblast-like morphology. At approximately 16 days in culture, hUCB-MSCs rapidly proliferated and appeared to be relatively uniform, exhibiting long spindle-like structures and colony distribution (Fig. 1A). Once the cells had grown to 70‑80% confluence, they were harvested and inoculated in passage culture flasks. After 3–5 passages, hUCB-MSCs showed a homogeneous fibroblast-like appearance and formed whirlpool-like clusters when they grew to 70% confluence (Fig. 1B).

After four passages of expansion and purification, both cell lines were assayed for multilineage differentiation potential. After 3 weeks of induction, hUCB-MSCs were able to differentiate into mature osteocytes, as confirmed by Alizarin Red S staining of calcium deposits (Fig. 1C), and mature adipocytes, as confirmed by Oil Red O staining of intracellular lipid vacuoles (Fig. 1D).

Flow cytometry analysis of the fourth passage of cultured hUCB-MSCs was performed to confirm immune phenotypes. The majority of expanded cells were positive for CD73 (99.80 ± 1.3%), CD90 (99.47 ± 0.9%), and CD105 (97.70 ± 1.8%) and were negative for CD34 (0.09 ± 0.05%) and HLA-DR (0.20 ± 0.09%) (Fig. 2). These results indicate that the cultured cells have a MSC phenotype, but lack specific haematopoietic and monocyte-macrophage antigen phenotypes. Therefore, we concluded that the adherent cells derived from hUCB were MSCs.

Figure 1


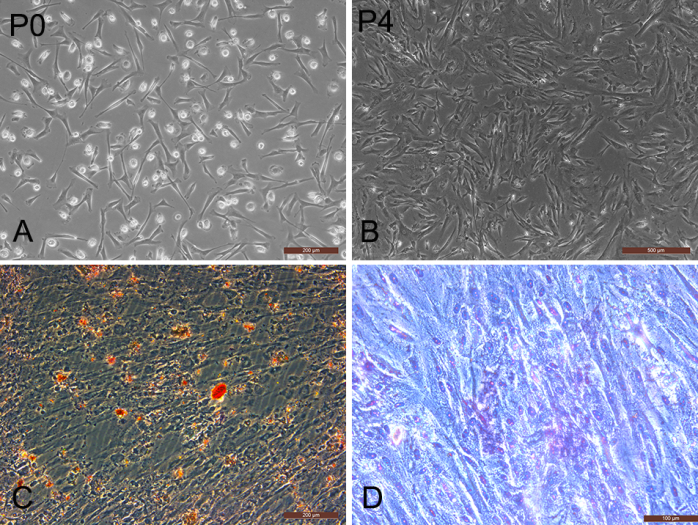


Fig. S1. Purification and differentiation of hUCB-MSCs in vitro. Stem cells from human umbilical cord blood were cultured from primary (A) to passage 4 (B). We noted that hUCB-MSCs of passage 4 displayed a homogeneous fibroblast-like appearance. hUCB-MSCs differentiated into osteocytes, as indicated by positive staining for calcium deposits with Alizarin Red S (C), and adipocytes, as indicated by positive staining for lipid vacuoles with Oil Red O (D).

Figure 2


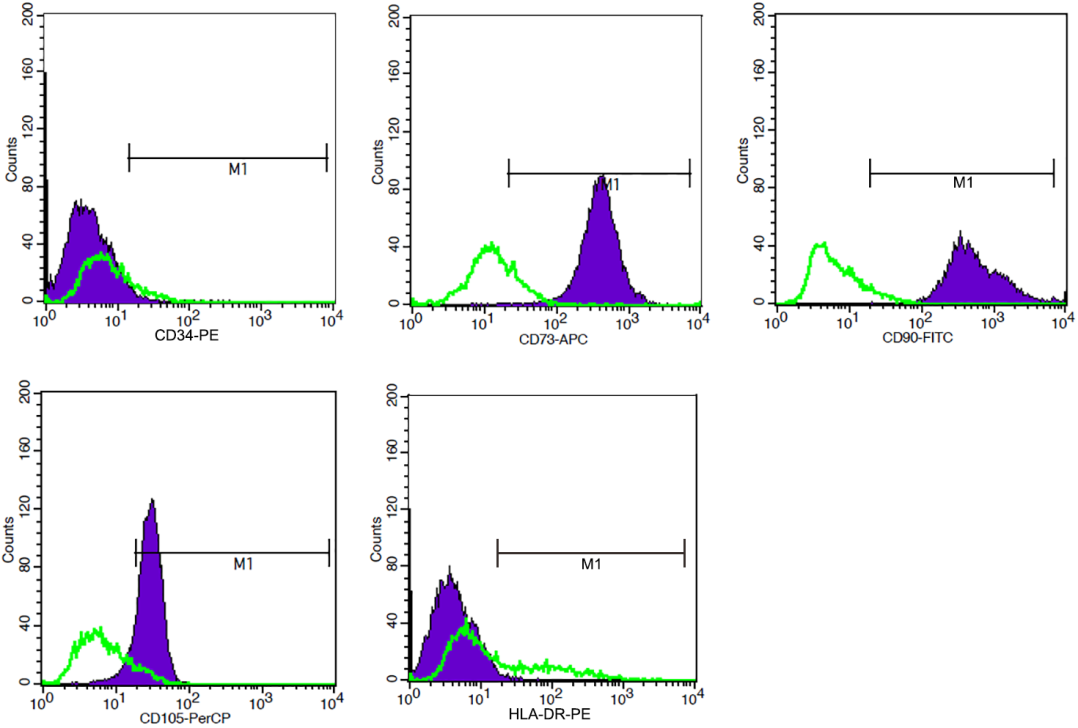


Fig. S2. Immunophenotype of hUCB-MSCs. The cells at passage 4 were trypsinised, labelled with antibodies against the indicated antigens, and analysed by flow cytometry. Green curves show isotype controls and blue curves show tested samples. The lack of CD34 and HLA-DR and the positive expression of CD73, CD90 and CD105 indicate mesenchymal stem cell lineage.
